# Supplementary material for: Measurement of violence against women and disability: protocol for a scoping review
Source: BMJ Open. 2020 Dec 23;10(12):e040104. doi: 10.1136/bmjopen-2020-040104 (PMC7768952; doi:10.1136/bmjopen-2020-040104)

BMJ Open is committed to open peer review. As part of this commitment we make the peer review history of every article we publish publicly available.

When an article is published we post the peer reviewers' comments and the authors' responses online. We also post the versions of the paper that were used during peer review. These are the versions that the peer review comments apply to.

The versions of the paper that follow are the versions that were submitted during the peer review process. They are not the versions of record or the final published versions. They should not be cited or distributed as the published version of this manuscript.

BMJ Open is an open access journal and the full, final, typeset and author-corrected version of record of the manuscript is available on our site with no access controls, subscription charges or pay-per-view fees (http://bmjopen.bmj.com).

If you have any questions on BMJ Open's open peer review process please email info.bmjopen@bmj.com

BMJ Open

Measurement of violence against women and disability: protocol for a scoping review

Journal:	*BMJ Open*
Manuscript ID	bmjopen-2020-040104
Article Type:	Protocol
Date Submitted by the Author:	06-May-2020
Complete List of Authors:	Meyer, Sarah; World Health Organization, Department of Reproductive Health and Research<br>Lasater, Molly; Johns Hopkins University Bloomberg School of Public Health, Mental Health<br>Lee, Lindsay; WHO, NVI/BDD; WHO<br>Garcia-Moreno, Claudia; World Health Organisation, Department of Sexual and Reproductive Health and Research
Keywords:	PUBLIC HEALTH, STATISTICS & RESEARCH METHODS, SEXUAL MEDICINE

SCHOLARONE™
Manuscripts

BMJ

I, the Submitting Author has the right to grant and does grant on behalf of all authors of the Work (as defined in the below author licence), an exclusive licence and/or a non-exclusive licence for contributions from authors who are: i) UK Crown employees; ii) where BMJ has agreed a CC-BY licence shall apply, and/or iii) in accordance with the terms applicable for US Federal Government officers or employees acting as part of their official duties; on a worldwide, perpetual, irrevocable, royalty-free basis to BMJ Publishing Group Ltd ("BMJ") its licensees and where the relevant Journal is co-owned by BMJ to the co-owners of the Journal, to publish the Work in this journal and any other BMJ products and to exploit all rights, as set out in our licence.

The Submitting Author accepts and understands that any supply made under these terms is made by BMJ to the Submitting Author unless you are acting as an employee on behalf of your employer or a postgraduate student of an affiliated institution which is paying any applicable article publishing charge ("APC") for Open Access articles. Where the Submitting Author wishes to make the Work available on an Open Access basis (and intends to pay the relevant APC), the terms of reuse of such Open Access shall be governed by a Creative Commons licence – details of these licences and which Creative Commons licence will apply to this Work are set out in our licence referred to above.

Other than as permitted in any relevant BMJ Author's Self Archiving Policies, I confirm this Work has not been accepted for publication elsewhere, is not being considered for publication elsewhere and does not duplicate material already published. I confirm all authors consent to publication of this Work and authorise the granting of this licence.

For peer review only

Measurement of violence against women and disability: protocol for a scoping review

Sarah R. Meyer[1], Molly E. Lasater[2], Lindsay Lee[3], Claudia García-Moreno[1]

1. Department of Sexual and Reproductive Health and Research, World Health Organization, Geneva, Switzerland.
2. Department of Mental Health, Johns Hopkins School of Public Health, Baltimore, USA.
3. Department of Noncommunicable Diseases, World Health Organization, Geneva, Switzerland.

Corresponding author: Dr Claudia García-Moreno, Department of Sexual and Reproductive Health and Research, World Health Organization, garciamorenoc@who.int, Phone number: 0041 22 791 4353, Fax number: 0041 22 791 4171

Key words: violence against women, systematic review, qualitative research, disability

Word count: 2958

For peer review only

For peer review only

Measurement of violence against women and disability: protocol for a scoping review

Sarah R. Meyer[1], Molly E. Lasater[2], Lindsay Lee[3], Claudia García-Moreno[1]

1. Department of Sexual and Reproductive Health and Research, World Health Organization, Geneva, Switzerland.
2. Department of Mental Health, Johns Hopkins School of Public Health, Baltimore, USA.
3. Department of Noncommunicable Diseases, World Health Organization, Geneva, Switzerland.

Corresponding author: Dr Claudia García-Moreno, Department of Sexual and Reproductive Health and Research, World Health Organization, garciamorenoc@who.int, Phone number: 0041 22 791 4353, Fax number: 0041 22 791 4171

Key words: violence against women, systematic review, qualitative research, disability

Word count: 2958

For peer review only

Abstract:

Introduction: Violence against women is a serious threat to women's health and human rights globally. Disability has been associated with increased risk of exposure to different forms of violence, however, there are questions concerning how best to measure this association. Research on understanding the association between violence and disability amongst women has included incorporating short disability measures into violence against women prevalence surveys. The potential to improve understanding of interconnections between violence and disability by measuring violence within disability-focused research is underexplored. The scoping review described here focuses on measurement of violence within the context of disability-focused research, research focused on the intersection of disability and violence, or measurement of disability in the context of research focused on violence against women. Specifically, we aim to map definitions, measures and methodologies used to measure violence against women, in the context of disability-focused research, and vice-versa.

Methods and analysis: For our scoping review, we will conduct searches for quantitative studies of disability-focused research which utilize measures of violence against women, and measures of disability in research focused on violence against women, in 11 online databases. We will search for grey literature, search the websites of National Statistics Offices for all countries to identify any national or sub-national disability research, and consult with experts for input. Two authors will independently review titles and abstracts retrieved through the search strategy. Data extraction will be conducted independently by one author and data will be analysed and synthesised using a thematic synthesis approach.

Ethics and dissemination: Ethics approval was not sought as no primary data is being collected. Findings will be disseminated through a publication in a peer-reviewed journal, through coordinated dissemination to researchers, practitioners, data users and generators, and through various working groups and networks on violence against women and disability.

Strengths and limitations of this study:

- This scoping review is designed with a comprehensive search strategy, including a structured search strategy for country-level and regional data that are unpublished in peer-reviewed literature;
- This scoping review focuses on a significant gap in the evidence, and provides an approach to mapping and understanding available measurement methods of

violence against women utilized in studies of disability and measurement methods of disability utilized in studies of violence against women;

- This scoping review uses appropriate search strategy, data extraction and analysis to comprehensively map the field of measurement of violence against women with disability

For peer review only

Violence against women is a serious threat to women's health and human rights globally. An expanding evidence-base has identified a number of global risk factors for women's exposure to violence, such as economic factors, including poverty, patterns of asset ownership and wealth inequalities [1], social norms concerning male authority over female behavior and norms justifying violence against women [2], and exposure to childhood abuse and exposure to intimate partner/domestic violence of one's mother as a child [3].

A potential risk factor that is currently poorly understood is disability. In particular, while it is hypothesized that disability may increase women's vulnerability to violence (and violence can also lead to disability), there is limited evidence concerning the intersection between disability and violence against women. A systematic review and meta-analysis of prevalence and risk of violence against adults with disabilities found that adults with disabilities are at increased risk of violence compared to adults without disability. However, all data were from studies conducted in high and middle-income contexts, and the review did not conduct sex-stratified analyses to identify if gender dimensions compounds the risk of violence against persons with disabilities [4].

Disability may be a risk factor for exposure to violence against women for a range of reasons. Studies have suggested that violence against women with disabilities is greater than violence against women without disabilities due to perpetrator-related characteristics. For example, women with disabilities are more likely to have partners who hold views supporting patriarchal dominance, and to be possessive and jealous, leading to enacting violence within the context of intimate partner relationships[5]. Qualitative studies have identified specific vulnerabilities to violence experienced by women with disabilities, including, reliance on partners for support in daily activities and/or in financial support, lack of social support and lack of availability of accessible services for prevention and response for women with disabilities experiencing violence

For peer review only

[6]. However, the evidence-base concerning disability as a risk factor for women's experience of violence is relatively limited.

Some existing reviews have addressed the intersection of disability and violence. A systematic review of studies exploring the intersection of violence and intellectual disabilities identified five qualitative and one mixed-methods study, concluding that the evidence is extremely sparse, and that "the current state of knowledge concerning the use and experience of partner violence by adults with intellectual disabilities is fundamentally inadequate, and until this knowledge gap is closed, our ability to provide appropriate evidence-based services to both perpetrators and victims is limited" [7]. A literature review focused on prevalence of interpersonal violence against persons with disabilities found widely varying study designs and definitions and measures of disability and violence. However, the data indicated consistent associations of disability with a higher exposure to lifetime and past 5-year intimate partner violence amongst women [8]. Women's experience of violence was not a specific focus in all of these reviews. A literature review of qualitative and quantitative studies addressing the question of prevalence and risk factors for violence amongst women with acquired disabilities, identified specific risk factors such as physical, economic and emotional dependency, and explored the role of social isolation in vulnerability to violence amongst women with disabilities [9].

A significant challenge in understanding disability as a risk factor for women's experience of violence is how to quantify this association, both in terms of measures of disability and of violence. Furthermore, disability can also be a consequence of intimate partner or other forms of violence against women, and the relationship between violence and disability may be bi-directional. Several issues have been identified with disability assessment tools. Different models of disability are linked to different measurement approaches [10]. Studies of disability globally employ vastly different definitions of disability or cut-offs to determine disability status across studies,

For peer review only

impacting prevalence estimates and comparability of data sources [11]. There are some measures, such as the Washington Group Short Set of Questions on Disability [Washington Group Questions], that have specifically been developed to address issues of comparability. They reflect an approach that assesses functioning, and whether persons with disability are able to participate in society[12]. However, research has indicated that the Washington Group questions do not reliably identify individuals who screen positive clinically for moderate or severe impairment [13]. Use of the Washington Questions for screening has been found to define individuals with mild to moderate disability as non-disabled [14].

Some recent efforts to understand how violence against women and disability intersect, and whether disability is a risk factor for exposure to violence against women, have focused on measuring disability within the context of violence against women prevalence studies or intervention studies. For example, the *What Works to Prevent Violence against Women and Girls Global Programme* included the Washington Group Questions in all quantitative impact assessments. Analyses from baseline assessments for interventions in six countries (Afghanistan, Bangladesh, Ghana, Nepal, South Africa, and Tajikistan) showed that women with disabilities are between two to four times more likely to experience intimate partner violence than women without disabilities [15].

Population-based prevalence studies of violence against women are a primary way of generating data on prevalence of, risk factors for and health outcomes of violence against women, and several recent studies of prevalence of violence against women have utilized the Washington Group questions to assess disability as a risk factor for exposure to violence [16]. For example, findings from the 2017 violence against women prevalence study in Mongolia indicate that ever-partnered women with moderate or severe disabilities reported higher lifetime physical and/or sexual intimate partner violence than women who reported no disability (no disability: 28.7%; moderate disability: 35.9%; severe disability: 44.7%) [17]. However, there are methodological and

For peer review only

practical challenges to using violence-focused prevalence studies to understand how disability is associated with violence. Cross-sectional prevalence studies do not enable assessment of whether self-reported disability is a risk factor for greater exposure to violence, or whether increased exposure to violence leads to higher (self-reported) disability amongst women. Sampling strategies in violence against women prevalence studies are household-based, and therefore exclude women with disabilities who may be living in other settings (i.e. institutions, group housing). Women with profound and severe disabilities are usually excluded from violence against women prevalence surveys, and there are challenging ethics concerns regarding interviewing women with specific disabilities that may impair communication or cognition [18]. In addition, some evidence indicates that women with disabilities may be exposed to different forms of violence and perpetrators than are traditionally captured in violence-focused research [15 19 20]. Therefore, measurement instruments presently utilized in violence-focused research may not adequately capture the range of types and perpetrators of violence against women with disabilities.

Aim:

This scoping review seeks to strengthen/support efforts to understand the linkages and intersections between disability and violence against women. Our scoping review focuses on measurement of violence within the context of disability-focused research, research focused on the intersection of disability and violence, and measurement of disability within research on violence against women. For the purpose of our review, we define disability-focused research as quantitative research seeking to estimate the prevalence of disability or identify associations between disability and other health outcomes. We define research focused on the intersection of disability and violence as research that focuses on associations between disability and violence, without being solely focused on either disability or violence as an outcome. Specifically, we aim to map definitions, measures and methodologies used to measure violence against women, in the context of disability-focused research.

For peer review only - http://bmjopen.bmj.com/site/about/guidelines.xhtml

Methods:

We will conduct a scoping review of studies published in peer-reviewed literature, and grey literature, including studies conducted or published by national statistical offices, World Health Organization and other international agencies. A scoping review is the most appropriate review method for studies that have exploratory research questions [21]. For this review, we seek to broadly map the field of measurement of violence against women, identify measures used, research gaps and explore feasibility of developing research objectives for a systematic review [22].

Search strategy

We identified the following domains as part of the research question: disability; women; violence; and quantitative research. For each of these domains, we identified the relevant keywords and search terms, which vary by database [see Table 1]. The search strategy will be appropriately modified for each database, including syntax and specific terms, topics and/ or headings. The search has not been limited by year of publication or type of publication.

Data sources

Data sources for the searches included following electronic databases: PubMed, PsycINFO, Embase, CINALH, Web of Science, PILOTS, Sociological Abstracts, ERIC, AgeLine, Social Work Abstracts, International Bibliography of the Social Sciences, Social Services Abstracts, ProQuest Criminal Justice, ASSIA, Dissertations & Theses Full Text, and Dissertations & Theses Global. We will also conduct Google searches for grey literature. We will search the websites of National Statistics Offices for all countries to identify any national or sub-national disability research. We will also review data and reports on disability available to the World Health Organization, which includes data from the WHO Model Disability Survey. We will identify 8-10 experts in the field of research on violence and/ or disability measurement, including researchers,

For peer review only

practitioners and policy makers, and contact them to provide any relevant literature. All experts will be contacted at least twice to provide the research team with additional resources to consider for inclusion. We will review the reference list of existing relevant systematic reviews and scoping reviews to identify relevant publications.

Inclusion and exclusion criteria

Studies will be eligible for this scoping review if the study:

i) Utilizes a quantitative methodology; mixed methods studies will be included if the quantitative data are reported separately; and
ii) Compares women with disability to women without disability (studies including men and women with disability will be included if sex-specific analyses are included) OR includes only women with disability; and
iii) Assesses exposure to any form of violence; and
iv) Examines violence experienced as an adult, aged 15 and older (studies including violence experienced before the age of 15 will be included if violence experienced above 15 is also measured).

There are no date limits. Non-English language articles will be included depending on number and capacity of team

Studies will not be eligible for this scope review if the study only:

i) Focuses only on mental health disorders (depression, anxiety, post-traumatic stress disorder); or
ii) Compares women with disability to men with disability; or
iii) Only focuses on violence experienced before the age of 15; or
iv) Utilizes data from case studies or client files;
v) Is based on caregiver report and/ or forensic exam;
vi) Focuses on validity/ reliability of the measure or scale development.

For peer review only

While mental disorders are often considered a part of disability, there is a wide literature specifically on the associations between violence and mental health, and these will be explored in a separate systematic review.

We will identify characteristics of studies (published and grey literature) meeting inclusion criteria, with a focus on mapping and evaluating measures of violence used in this research, identifying types of violence assessed, instruments utilized, and specifics of measures (i.e. perpetrator, time frame). This review differs from previous reviews of violence against persons with disability by focusing on: i) women, ii) any setting (community, institution, for example), iii) any type of violence and perpetrator, iv) measurement of disability (which measures, how measured), and iv) measurement of violence (which measures, how measured).

Data management

EndNote V.X9 will be used as our bibliographic software management platform. We will remove duplicates using EndNote, prior to exporting titles and abstracts to an Excel spreadsheet for review. Data extraction results will be recorded in separate Excel spreadsheets. A flow diagram will be presented in any final publications, showing results of each stage of the review and adhering to the PRISMA statement.

Selection of studies

Two authors will independently review titles and abstracts retrieved through the search strategy, to determine which should be included for full text review. If an abstract or title is considered relevant by either of the authors, it will be included for full text review. Two authors will independently review all articles selected for full text review for eligibility, to reach consensus on inclusion in the review. Any discrepancies will be resolved with the input of the third team member. Reasons for excluding articles will be recorded.

For peer review only

Data extraction

After full text review, the following data will be extracted from all included articles using a standardized data extraction form: country studied; research questions; study design (comparing individuals with disability vs. people without disability); sampling method and sample characteristics; data collection (measurement method); disability measurement (definitions, measurement [self-report, instrument], measure of severity, functional impairment); violence measurement (definition, types measured, perpetrators, time frame, instruments use); data analysis methods; risk and protective factors; main findings (as reported by the study's own authors); ethical considerations and discussion of disability and violence specific issues; and any reported study limitations.

Data extraction will be conducted independently by one author (SM), and accuracy of the data extraction checked by a second author, with discrepancies resolved by discussion and, if necessary, by discussion with another author (CGM) to reach consensus. Reviewers will develop and pre-test a data extraction spreadsheet, to be used to compile a summary of characteristics and key findings of the included studies. The spreadsheet will also include categories relevant to data synthesis, described further below. We will not conduct quality assessment, given this is a scoping review.

Data synthesis

We will present results of the search and data extraction, using both simple quantitative summaries (i.e. tabulation of % of studies from each region, % of studies that utilized specific sampling methods), and narrative synthesis of the studies, which includes highlighting similarities and differences in the measures of disability and of violence employed in the included studies, and exploration of other patterns in aspects of study design and measurement methodologies in included studies [23].

Patient and public involvement:

For peer review only

Patients were not involved in the development of this scoping review. Public were not consulted specifically for the development of the research questions, however, previous research and consultations with experts has indicated that this is a relevant and important area of enquiry in the field of violence against women research.

Discussion:

This manuscript describes a protocol for a scoping review of measurement of violence against women within the context of disability-focused research and vice versa. Strengths of the review include a rigorous and expansive search strategy, including disability and violence against women studies conducted by National Statistical Offices and not published in peer-reviewed literature; a clear and structured process of data extraction; and a focus on generating a map of available measures and methodologies assessing the intersection of violence and disability, within a body of evidence that has not been rigorously reviewed. This will contribute to the discussion on improving the ways of measuring the intersections of disability and violence against women.

A primary limitation is the definition of disability that is operationalized in the review. The World Health Organization recognizes that disability includes “impairments, activity limitations, and participation restrictions,” and that disability “is a complex phenomenon, reflecting the interaction between features of a person’s body and features of the society in which he or she lives” (https://www.who.int/topics/disabilities/en/). According to this definition, knowledge of the environment in which a person lives is critical to understanding someone’s experience of disability. However, to keep the scope of the review manageable and with the understanding that research on the environmental component of disability is often lacking, we will utilize search terms for disability that primarily focus on impairments or specific health conditions that are known to cause particular impairments. This may limit the literature identified and included in the search and bias results towards research focused on only the bodily, health or impairment component of disability.

For peer review only

Improved understanding and expanded evidence on how disability and violence against women intersect is needed to develop effective evidence-based programming and policy for prevention and response to violence against women globally. Various aspects of lived experiences of disability may influence types of violence experienced, access to and utilization of services, and ways in which research, policy and programming on violence against women can be adapted or refined to adequately address the needs of women with disability who experience violence. Yet, as a report from a recent expert consultation on measurement of violence against women with disabilities noted, “far more work needs to be done to establish appropriate, effective, agreed and internationally comparable methods for measuring many of the structural, institutional and interpersonal forms of violence that women with disabilities experience on a daily basis”[18]. Expanding understanding of linkages and intersections between disability and violence against women requires further consideration of how disability and violence are currently being assessed, including what type(s) of measures are being used and within what type(s) of methodologies and study designs. This scoping review will establish how violence is measured within disability-focused research, and how research on violence against women measures disability. Findings of the review will be used to inform recommendations regarding evidence-generation on disability and violence against women.

Ethics and dissemination:

Findings will be with researchers, practitioners, data users and generators with an interest in violence against women, and experts selected to participate in a working group on disability within the Technical Advisory Group to the Interagency Working Group on Violence against Women Estimation and Data, a group of experts on measurement of violence against women and global violence against women data convened by the Department of Sexual and Reproductive Health and Research, WHO. Final outcomes will be presented in a manuscript for publication in a peer-reviewed

For peer review only

journal. This will be disseminated through the Interagency Working Group above and other partners. The Sensory Functions, Disability and Rehabilitation Team will also disseminate through their networks focused on disability, including through the Interagency Support Group for the Convention on the Rights of Persons with Disabilities.

For peer review only

Contribution: SM and CGM designed and developed the scoping review, which was conceptualized by CGM. SM, ML and LL developed and refined search strategies with input from CGM. SM prepared the manuscript with substantive input from all other authors. All authors reviewed the manuscript prior to submission.

Competing interest: There are no competing interests.

Funding: The scoping review will be conducted with funding from the Department for International Development for the UN Women-World Health Organization Joint Programme on Strengthening Methodologies and Measurement and building national capacities for violence against women data through the UNDP-UNFPA-UNICEF-WHO-World Bank Special Programme of Research, Development and Research Training in Human Reproduction (HRP), a cosponsored programme executed by the World Health Organization (WHO).

For peer review only

1. Gibbs A, Duvvury N, Scriver S. What Works Evidence Review: The relationship between poverty and intimate partner violence. In: What Works to Prevent Violence, ed., 2017.
2. Heise LL, Kotsadam A. Cross-national and multilevel correlates of partner violence: an analysis of data from population-based surveys. *The Lancet Global health* 2015;3(6):e332-40. doi: 10.1016/s2214-109x(15)00013-3 [published Online First: 2015/05/24]
3. Abramsky T, Watts CH, Garcia-Moreno C, et al. What factors are associated with recent intimate partner violence? findings from the WHO multi-country study on women's health and domestic violence. *BMC public health* 2011;11:109. doi: 10.1186/1471-2458-11-109 [published Online First: 2011/02/18]
4. Hughes K, Bellis MA, Jones L, et al. Prevalence and risk of violence against adults with disabilities: a systematic review and meta-analysis of observational studies. *Lancet (London, England)* 2012;379(9826):1621-9. doi: 10.1016/s0140-6736(11)61851-5 [published Online First: 2012/03/02]
5. Brownridge DA, Ristock J, Hiebert-Murphy D. The high risk of IPV against Canadian women with disabilities. *Medical science monitor : international medical journal of experimental and clinical research* 2008;14(5):Ph27-32. [published Online First: 2008/04/30]
6. Copel LC. Partner abuse in physically disabled women: a proposed model for understanding intimate partner violence. *Perspectives in psychiatric care* 2006;42(2):114-29. doi: 10.1111/j.1744-6163.2006.00059.x [published Online First: 2006/05/09]
7. Bowen E, Swift C. The Prevalence and Correlates of Partner Violence Used and Experienced by Adults With Intellectual Disabilities: A Systematic Review and Call to Action. *Trauma, violence & abuse* 2017:1524838017728707. doi: 10.1177/1524838017728707 [published Online First: 2018/01/16]
8. Hughes RB, Lund EM, Gabrielli J, et al. Prevalence of interpersonal violence against community-living adults with disabilities: a literature review. *Rehabilitation psychology* 2011;56(4):302-19. doi: 10.1037/a0025620 [published Online First: 2011/11/30]
9. Plummer SB, Findley PA. Women with disabilities' experience with physical and sexual abuse: review of the literature and implications for the field. *Trauma, violence & abuse* 2012;13(1):15-29. doi: 10.1177/1524838011426014 [published Online First: 2011/11/11]
10. Mont D. Measuring health and disability. *Lancet (London, England)* 2007;369(9573):1658-63. doi: 10.1016/s0140-6736(07)60752-1 [published Online First: 2007/05/15]
11. Trani JF, Babulal GM, Bakhshi P. Development and Validation of the 34-Item Disability Screening Questionnaire (DSQ-34) for Use in Low and Middle Income Countries Epidemiological and Development Surveys. *PloS one* 2015;10(12):e0143610. doi: 10.1371/journal.pone.0143610 [published Online First: 2015/12/03]
12. Madans JH, Loeb M. Methods to improve international comparability of census and survey measures of disability. *Disability and rehabilitation*

2013;35(13):1070-3. doi: 10.3109/09638288.2012.720353 [published Online First: 2012/10/02]
13. Mactaggart I, Kuper H, Murthy GV, et al. Measuring Disability in Population Based Surveys: The Interrelationship between Clinical Impairments and Reported Functional Limitations in Cameroon and India. *PloS one* 2016;11(10):e0164470. doi: 10.1371/journal.pone.0164470 [published Online First: 2016/10/16]
14. Sabariego C, Oberhauser C, Posarac A, et al. Measuring Disability: Comparing the Impact of Two Data Collection Approaches on Disability Rates. *International journal of environmental research and public health* 2015;12(9):10329-51. doi: 10.3390/ijerph120910329 [published Online First: 2015/08/27]
15. Dunkle K, van der Heijden I, Stern E, et al. Disability and Violence against Women and Girls: Emerging Evidence from the What Works to Prevent Violence against Women and Girls Global Programme. In: Works W, ed., 2018.
16. The Asia Foundation. Understanding violence against women and children in Timor-Leste: Findings from the Nabilan Baseline Study – Main Report. Dili The Asia Foundation, 2016.
17. UNFPA, National Statistics Office of Mongolia. 2017 National Study on Gender-based Violence in Mongolia. Ulaanbaatar, 2018.
18. Vaughan C, Devine A, Diemer K, et al. Measuring the Prevalence of Violence against Women with Disabilities: kNOwVAWdata Initiative, 2018.
19. Vaughan C, Devine A, Ignacio R. Building capacity for a disability-inclusive response to violence against women and girls: experiences from the W-DARE project in the Philippines. *Gender & Development* 2016;24(2):245-60.
20. Shah S, Tsitsou L, Woodin S. Hidden Voices: Disabled Women's Experiences of Violence and Support Over the Life Course. *Violence against women* 2016;22(10):1189-210. doi: 10.1177/1077801215622577 [published Online First: 2016/01/15]
21. Colquhoun HL, Levac D, O'Brien KK, et al. Scoping reviews: time for clarity in definition, methods, and reporting. *Journal of clinical epidemiology* 2014;67(12):1291-4. doi: 10.1016/j.jclinepi.2014.03.013 [published Online First: 2014/07/19]
22. Arksey H, O'Malley L. Scoping studies: towards a methodological framework. *Int J Soc Res Methodol* 2005;8:19-32.
23. Ryan R. Cochrane Consumers and Communication Review Group: data synthesis and analysis: Cochrane Consumers and Communication Review Group 2013.

For peer review only

Table 1 – PubMed Search Strategy

1	"Intellectual disability"[MeSH] OR "Communication disorders"[MeSH] OR "Developmental disabilities"[MeSH] OR "Mentally Disabled persons"[MeSH] OR "Disabled persons"[MeSH] OR "physical disabilit*"[TIAB] OR "physically disabled"[TIAB] OR "intellectual disabilit*"[TIAB] OR "handicap"[TIAB] OR "functional impairment"[TIAB] OR "mental disorder*"[TIAB] OR "mentally disabled"[TIAB] OR "mental disability*"[TIAB]
2	Women[MeSH] OR female[MeSH] OR wife[TIAB] OR spouses[MeSH] OR wives[TIAB] OR "female partner*"[TIAB] OR spouse*[TIAB]
3	"Elder abuse"[MeSH] OR "domestic violence"[MeSH] OR "Intimate Partner Violence"[MeSH] OR "battered women"[MeSH] OR "violence"[MeSH] OR "aggression"[MeSH] OR "spouse abuse"[MeSH] OR "Physical Abuse"[MeSH] OR Rape [MeSH] OR "elder neglect"[TIAB] OR "elder mistreatment"[TIAB] OR "elder maltreatment"[TIAB] OR "assault"[TIAB] OR "sexual abuse"[TIAB] OR "sexual assault"[TIAB] OR "rape"[TIAB] OR "psychological abuse"[TIAB] OR "psychological violence"[TIAB] OR "emotional abuse"[TIAB] OR "emotional violence"[TIAB] OR "neglect"[TIAB] OR "economic abuse"[TIAB] OR "verbal abuse"[TIAB] OR "violence against women"[TIAB] OR "abused women"[tiab] OR "intimate terrorism"[tiab] OR "marital rape"[tiab] OR "wife beating"[tiab] OR "relationship aggression"[tiab]
4	"epidemiologic methods"[MeSH] OR "Comparative Study"[Publication Type] OR "outcome and process assessment (health care)"[Mesh] OR "statistics and numerical data"[Subheading] OR "Evaluation Studies"[Publication Type] OR "meta analysis"[Publication Type] OR "multicenter study"[Publication Type] OR "incidence"[TIAB] OR "surveillance"[TIAB] OR "prevalence"[TIAB] OR "epidemiology"[subheading] OR "Health Care Evaluation Mechanisms"[Mesh] OR "morbidity"[TIAB] OR "burden"[TW] OR "Cross sectional study"[MeSH] OR "case-control studies"[MeSH] OR "Cohort studies"[MeSH] OR "Surveys and questionnaires"[MeSH] OR "cross-sectional stud*"[TIAB] OR "quantitative survey"[TIAB] OR "survey"[TIAB]
1 AND 2 AND 3 AND 4	

For peer review only

BMJ Open

Measurement of violence against women and disability: protocol for a scoping review

Journal:	*BMJ Open*
Manuscript ID	bmjopen-2020-040104.R1
Article Type:	Protocol
Date Submitted by the Author:	15-Aug-2020
Complete List of Authors:	Meyer, Sarah; World Health Organization, Department of Reproductive Health and Research<br>Lasater, Molly; Johns Hopkins University Bloomberg School of Public Health, Mental Health<br>Lee, Lindsay; WHO, NVI/BDD; WHO<br>Garcia-Moreno, Claudia; World Health Organisation, Department of Sexual and Reproductive Health and Research
**Primary Subject Heading**:	Public health
Secondary Subject Heading:	Global health
Keywords:	PUBLIC HEALTH, STATISTICS & RESEARCH METHODS, SEXUAL MEDICINE

SCHOLARONE™ Manuscripts

I, the Submitting Author has the right to grant and does grant on behalf of all authors of the Work (as defined in the below author licence), an exclusive licence and/or a non-exclusive licence for contributions from authors who are: i) UK Crown employees; ii) where BMJ has agreed a CC-BY licence shall apply, and/or iii) in accordance with the terms applicable for US Federal Government officers or employees acting as part of their official duties; on a worldwide, perpetual, irrevocable, royalty-free basis to BMJ Publishing Group Ltd ("BMJ") its licensees and where the relevant Journal is co-owned by BMJ to the co-owners of the Journal, to publish the Work in this journal and any other BMJ products and to exploit all rights, as set out in our licence.

The Submitting Author accepts and understands that any supply made under these terms is made by BMJ to the Submitting Author unless you are acting as an employee on behalf of your employer or a postgraduate student of an affiliated institution which is paying any applicable article publishing charge ("APC") for Open Access articles. Where the Submitting Author wishes to make the Work available on an Open Access basis (and intends to pay the relevant APC), the terms of reuse of such Open Access shall be governed by a Creative Commons licence – details of these licences and which Creative Commons licence will apply to this Work are set out in our licence referred to above.

Other than as permitted in any relevant BMJ Author's Self Archiving Policies, I confirm this Work has not been accepted for publication elsewhere, is not being considered for publication elsewhere and does not duplicate material already published. I confirm all authors consent to publication of this Work and authorise the granting of this licence.

For peer review only

Measurement of violence against women and disability: protocol for a scoping review

Sarah R. Meyer[1], Molly E. Lasater[2], Lindsay Lee[3], Claudia García-Moreno[1]

1. Department of Sexual and Reproductive Health and Research, World Health Organization, Geneva, Switzerland.
2. Department of Mental Health, Johns Hopkins School of Public Health, Baltimore, USA.
3. Department of Noncommunicable Diseases, World Health Organization, Geneva, Switzerland.

Corresponding author: Dr Claudia García-Moreno, Department of Sexual and Reproductive Health and Research, World Health Organization, garciamorenoc@who.int, Phone number: 0041 22 791 4353, Fax number: 0041 22 791 4171

Key words: violence against women, systematic review, qualitative research, disability

Word count: 4572

For peer review only

Abstract:

Introduction: Violence against women is a serious threat to women's health and human rights globally. Disability has been associated with increased risk of exposure to different forms of violence, however, there are questions concerning how best to measure this association. Research on understanding the association between violence and disability amongst women has included incorporating short disability measures into violence against women prevalence surveys. The potential to improve understanding of interconnections between violence and disability by measuring violence within disability-focused research is underexplored. The scoping review described here focuses on three areas of measurement of violence against women and disability: i) measurement of violence within the context of disability-focused research, ii) measurement in research focused on the intersection of disability and violence, and iii) measurement of disability in the context of research focused on violence against women. Specifically, we aim to map definitions, measures and methodologies in these areas, globally.

Methods and analysis: For our scoping review, we will conduct searches for quantitative studies of disability-focused research which utilize measures of violence against women, and measures of disability in research focused on violence against women, in 11 online databases. Two authors will independently review titles and abstracts retrieved through the search strategy. We will search for grey literature, search the websites of National Statistics Offices for all countries to identify any national or sub-national disability research, and consult with experts for input. Data extraction will be conducted independently by one author and reviewed by another author, and data will be analysed and synthesised using a thematic synthesis approach.

Ethics and dissemination: Ethics approval was not sought as no primary data is being collected. Findings will be disseminated through a publication in a peer-reviewed journal, through coordinated dissemination to researchers, practitioners, data users and generators, and through various working groups and networks on violence against women and disability.

Strengths and limitations of this study:

- This scoping review is designed with a comprehensive search strategy, including a structured search strategy for country-level and regional data that are unpublished in peer-reviewed literature;
- This scoping review focuses on a significant gap in the evidence, and provides an approach to mapping and understanding available measurement methods of

For peer review only - http://bmjopen.bmj.com/site/about/guidelines.xhtml

violence against women utilized in studies of disability and measurement methods of disability utilized in studies of violence against women;

- This scoping review uses appropriate search strategy, data extraction and analysis to comprehensively map the global field of measurement of violence against women with disability

For peer review only

Violence against women is a serious threat to women's health and human rights globally. The wide range of severe and often long-lasting physical and mental health impacts of violence against women constitute a global public health threat. An expanding evidence-base has identified a number of global risk factors for women's exposure to violence, such as economic factors, including poverty, patterns of asset ownership and wealth inequalities [1], social norms concerning male authority over female behavior and norms justifying violence against women [2], and exposure to childhood abuse and exposure to intimate partner/domestic violence of one's mother as a child [3].

A potential risk factor that is currently poorly understood is disability. In particular, while it is hypothesized that disability may increase women's vulnerability to violence (and violence can also lead to disability), there is limited evidence concerning the intersection between disability and violence against women. A systematic review and meta-analysis of prevalence and risk of violence against adults with disabilities found that adults with disabilities are at increased risk of violence compared to adults without disability. However, the review did not conduct sex-stratified analyses to identify if gender dimensions compounds the risk of violence against persons with disabilities [4]. Disability may be a risk factor for exposure to violence against women for a range of reasons. Studies have suggested that violence against women with disabilities is greater than violence against women without disabilities due to perpetrator-related characteristics. For example, women with disabilities are more likely to have partners who hold views supporting patriarchal dominance, and to be possessive and jealous, leading to enacting violence within the context of intimate partner relationships[5]. Qualitative studies have identified specific vulnerabilities to violence experienced by women with disabilities, including, reliance on partners for support in daily activities and/or for financial support, lack of social support and lack of availability of accessible services for violence prevention and response for women with disabilities [6]. However, the evidence-base concerning disability as a risk factor for women's experience of

For peer review only - http://bmjopen.bmj.com/site/about/guidelines.xhtml

violence is relatively limited. Target 5.2 of the Sustainable Development Goals is, "End all violence against and exploitation of women and girls," and the over-arching goal of the SDGs is to "leave no-one behind." Within the framework of the SDGs, there is a need for a strengthened evidence-base concerning violence against women and disability, to inform violence prevention and policy response to violence against women and ensure effective design and implementation of policies, services and programs [7]. Developing and strengthening this evidence-base requires rigorous study design and measurement, and this scoping review emerges from the need to improve understanding of appropriate and effective measures and methodologies to shed light on the intersection between disability and violence against women.

Some studies and reviews have addressed the question of disability as a risk factor for women's experiences of violence. For example, the *What Works to Prevent Violence against Women and Girls Global Programme* included the Washington Group Short Set of Questions on Disability [Washington Group Questions] in quantitative impact assessments. Analyses from baseline assessments for interventions in six countries (Afghanistan, Bangladesh, Ghana, Nepal, South Africa, and Tajikistan) showed that women with disabilities are between two to four times more likely to experience intimate partner violence than women without disabilities [8]. A systematic review of studies exploring the intersection of violence and intellectual disabilities identified five qualitative and one mixed-methods study, concluding that the evidence is extremely sparse, and that "the current state of knowledge concerning the use and experience of partner violence by adults with intellectual disabilities is fundamentally inadequate, and until this knowledge gap is closed, our ability to provide appropriate evidence-based services to both perpetrators and victims is limited" [9]. A literature review focused on prevalence of interpersonal violence against persons with disabilities found the data indicated consistent associations of disability with a higher exposure to lifetime and past 5-year intimate partner violence amongst women [10]. Women's experience of violence was not a specific focus in all of these reviews. A literature review of qualitative and

For peer review only - http://bmjopen.bmj.com/site/about/guidelines.xhtml

quantitative studies addressing the question of prevalence and risk factors for violence amongst women with acquired disabilities, identified specific risk factors such as physical, economic and emotional dependency, and explored the role of social isolation in vulnerability to violence amongst women with disabilities [11].

A significant challenge in understanding disability as a risk factor for women's experience of violence is how to quantify this association, both in terms of measures of disability and of violence. Disability can also be a consequence of intimate partner or other forms of violence against women, and the relationship between violence and disability may be bi-directional. For example, pre-existing disabilities may be a risk factor for violence victimization, and women's experiences of violence may lead to disability, which entails that the relationship between violence and disability can be difficult to disentangle. Further compounding this challenge are the issues with disability assessment tools. Different conceptual models of disability are linked to different measurement approaches [12]. Studies of disability globally employ vastly different definitions of disability or cut-offs to determine disability status across studies, impacting prevalence estimates and comparability of data sources [13]. There are some measures, such as Washington Group Questions, that have specifically been developed to address issues of comparability. They reflect an approach that assesses functioning, and whether persons with disability are able to participate in society[14]. However, research has indicated that the Washington Group questions do not reliably identify individuals who screen positive clinically for moderate or greater impairment [15]. Use of the Washington Questions for screening has been found to define individuals with mild to moderate clinical impairments as non-disabled [16].

Population-based prevalence studies of violence against women are a primary way of generating data on prevalence of, risk factors for and health outcomes of violence against women. Recent donor interest in and support of strengthening evidence concerning violence against women with disabilities has focused on incorporating

For peer review only - http://bmjopen.bmj.com/site/about/guidelines.xhtml

disability questions within population-based national violence against women prevalence surveys, for example, in Timor Leste and Mongolia [17 18]. However, there are methodological and practical challenges to using violence-focused prevalence studies to understand how disability is associated with violence. Cross-sectional prevalence studies do not enable assessment of whether self-reported disability is a risk factor for greater exposure to violence, or whether increased exposure to violence leads to higher (self-reported) disability amongst women. Sampling strategies in violence against women prevalence studies are household-based, and therefore exclude women with disabilities who may be living in other settings (i.e. institutions, group housing). Women with profound and severe disabilities are usually excluded from violence against women prevalence surveys, and there are challenging ethics concerns regarding interviewing women with specific disabilities that may impair communication or cognition [19]. In addition, some evidence indicates that women with disabilities may be exposed to different forms of violence and perpetrators than are traditionally captured in violence-focused research [8 20 21]. Therefore, measurement instruments presently utilized in violence-focused research may not adequately capture the range of types and perpetrators of violence against women with disabilities.

Aim:

This scoping review seeks to strengthen and support efforts to understand the linkages and intersections between disability and violence against women, specifically by mapping definitions, measures and methodologies in quantitative literature on this topic.

Violence against women is defined by the United Nations as "any act of gender-based violence that results in, or is likely to result in, physical, sexual or mental harm or suffering to women, including threats of such acts, coercion or arbitrary deprivation of liberty, whether occurring in public or in private life" [22]. The World Health Organization defines disability as described in the International Classification of Functioning, Disability

For peer review only - http://bmjopen.bmj.com/site/about/guidelines.xhtml

and Health [ICF]: disability is the outcome of the interaction between (1) one's health conditions and (2) contextual factors such as physical accessibility of the environment, access to assistive products, or attitudes of others. To describe a person's disability status under the ICF framework, it is necessary to examine both components of this definition [23].

Our scoping review will map definitions, measures and methodologies in three areas of measurement of violence against women and disability: i) measurement of violence within the context of disability-focused research, ii) measurement in research focused on the intersection of disability and violence, and iii) measurement of disability in the context of research focused on violence against women. We focus on quantitative literature given our scoping review emerges from data requirements for the SDGs and seeks to address current developments in quantitative population-based surveys of violence against women. For the purpose of our review, we define disability-focused research as quantitative research seeking to estimate the prevalence of disability or identify associations between disability and other health outcomes. We define research focused on the intersection of disability and violence as research that focuses on associations between disability and violence, without being solely focused on either disability or violence as an outcome. This focus on three distinct, but overlapping, areas of literature is designed to inform current debates and discussions regarding how to generate evidence concerning violence against women with disabilities. As noted above, for example, donors' interest in understanding the association between disability and violence against women has focused on incorporating measures of disability within national violence against women surveys. However, a broader characterization of which measures of disability and violence are used and available, how definitions are operationalized, and what methodologies are feasible and appropriate is needed.

Methods:

Arksey and O'Malley lay out a framework for methods of scoping reviews that we draw upon in design of our protocol [24].

For peer review only - http://bmjopen.bmj.com/site/about/guidelines.xhtml

We will conduct a scoping review of studies published in peer-reviewed literature, and grey literature, including studies conducted or published by national statistical offices, World Health Organization and other United Nations agencies. A scoping review is the most appropriate review method for studies that have exploratory research questions [25]. Scoping reviews, described as commonly used for "reconnaissance," are specifically useful in contexts "where a body of literature has not yet been comprehensively reviewed, or exhibits a large, complex or heterogeneous nature," as is the case in the body of literature in question in this scoping review [26]. Scoping reviews are typically useful for mapping a field "in terms of its nature, features and volume," and given the state of knowledge and existing available evidence syntheses, this is the most appropriate type of review to address our research questions. In contrast to a systematic review, which focuses on a specific question, or set of questions, with a more tightly limited field of enquiry, a scoping review takes a broader approach to focus on mapping the literature and clarifying key concepts, enabling greater breadth than a systematic review. For this review, we seek to map the field of measurement of violence against women and disability in different bodies of literature, identify measures used, research gaps and explore feasibility of developing research objectives for a systematic review [24].

The following sections on search strategy, data searches, inclusion and exclusion criteria and selection of studies correspond to Arksey and O'Malley's Stage 2, Identifying relevant studies, and Stage 3, Selection of studies.

Search strategy

We identified the following domains as part of the research question: disability; women; violence; and quantitative research. For each of these domains, we identified the relevant keywords and search terms, which vary by database [see Table 1]. The search strategy will be appropriately modified for each database, including syntax and specific

For peer review only - http://bmjopen.bmj.com/site/about/guidelines.xhtml

terms, topics and/ or headings. The search has not been limited by year of publication or type of publication. An expert librarian at the World Health Organization provided advice on search strategy and selection of databases.

For peer review only

For peer review only - http://bmjopen.bmj.com/site/about/guidelines.xhtml

Table 1 – PubMed Search Strategy

1	"Intellectual disability"[MeSH] OR "Communication disorders"[MeSH] OR "Developmental disabilities"[MeSH] OR "Mentally Disabled persons"[MeSH] OR "Disabled persons"[MeSH] OR "physical disabilit*"[TIAB] OR "physically disabled"[TIAB] OR "intellectual disabilit*"[TIAB] OR "handicap"[TIAB] OR "functional impairment"[TIAB] OR "mental disorder*"[TIAB] OR "mentally disabled"[TIAB] OR "mental disability*"[TIAB]
2	Women[MeSH] OR female[MeSH] OR wife[TIAB] OR spouses[MeSH] OR wives[TIAB] OR "female partner*"[TIAB] OR spouse*[TIAB]
3	"Elder abuse"[MeSH] OR "domestic violence"[MeSH] OR "Intimate Partner Violence"[MeSH] OR "battered women"[MeSH] OR "violence"[MeSH] OR "aggression"[MeSH] OR "spouse abuse"[MeSH] OR "Physical Abuse"[MeSH] OR Rape [MeSH] OR "elder neglect"[TIAB] OR "elder mistreatment"[TIAB] OR "elder maltreatment"[TIAB] OR "assault"[TIAB] OR "sexual abuse"[TIAB] OR "sexual assault"[TIAB] OR "rape"[TIAB] OR "psychological abuse"[TIAB] OR "psychological violence"[TIAB] OR "emotional abuse"[TIAB] OR "emotional violence"[TIAB] OR "neglect"[TIAB] OR "economic abuse"[TIAB] OR "verbal abuse"[TIAB] OR "violence against women"[TIAB] OR "abused women"[tiab] OR "intimate terrorism"[tiab] OR "marital rape"[tiab] OR "wife beating"[tiab] OR "relationship aggression"[tiab]
4	"epidemiologic methods"[MeSH] OR "Comparative Study"[Publication Type] OR "outcome and process assessment (health care)"[Mesh] OR "statistics and numerical data"[Subheading] OR "Evaluation Studies"[Publication Type] OR "meta analysis"[Publication Type] OR "multicenter study"[Publication Type] OR "incidence"[TIAB] OR "surveillance"[TIAB] OR "prevalence"[TIAB] OR "epidemiology"[subheading] OR "Health Care Evaluation Mechanisms"[Mesh] OR "morbidity"[TIAB] OR "burden"[TW] OR "Cross sectional study"[MeSH] OR "case-control studies"[MeSH] OR "Cohort studies"[MeSH] OR "Surveys and questionnaires"[MeSH] OR "cross-sectional stud*"[TIAB] OR "quantitative survey"[TIAB] OR "survey"[TIAB]
1 AND 2 AND 3 AND 4	

For peer review only - http://bmjopen.bmj.com/site/about/guidelines.xhtml

Data sources

Data sources for the searches included following electronic databases: PubMed, PsycINFO, Embase, CINAHL, Web of Science, PILOTS, Sociological Abstracts, ERIC, AgeLine, Social Work Abstracts, International Bibliography of the Social Sciences, Social Services Abstracts, ProQuest Criminal Justice, ASSIA, Dissertations & Theses Full Text, and Dissertations & Theses Global. The grey literature search will be conducted by one author (SM), who will conduct structured google searches: "Country X disability survey," "Country X disability study" and "Country X disability statistics," for each country, reviewing 10 pages of results per search.

We will search the websites of National Statistics Offices for all countries to identify any national or sub-national disability research, as well as national violence against women studies and Demographic and Health Surveys that have included both disability and violence against women modules. We will also review data and reports on disability available to the World Health Organization, which includes data from the WHO Model Disability Survey. We will identify experts in the field of research on violence and/ or disability measurement, including researchers, practitioners and policy makers, and contact them to provide any relevant literature. All experts will be contacted at least twice to provide the research team with additional resources to consider for inclusion. We will review the reference list of existing relevant systematic reviews and scoping reviews to identify relevant publications.

Inclusion and exclusion criteria

Studies will be eligible for this scoping review if the study:

i) Utilizes a quantitative methodology; mixed methods studies will be included if the quantitative data are reported separately; and
ii) Compares women with disability to women without disability (studies including men and women with disability will be included if sex-specific analyses are included) OR includes only women with disability; and

For peer review only - http://bmjopen.bmj.com/site/about/guidelines.xhtml

iii) Assesses exposure to any form of violence; and
iv) Examines violence experienced as an adult, aged 15 and older (studies including violence experienced before the age of 15 will be included if violence experienced above 15 is also measured).

15 and above is selected as the age cut-off as this is lower age-limit for relevant SDG indicators regarding elimination of violence against women and girls. Non-English language articles will be included depending on number and capacity of team, which includes members who are fluent in Spanish, French and Portuguese.

Studies will not be eligible for this scoping review if the study only:

i) Focuses only on common mental health disorders (depression, anxiety, post-traumatic stress disorder [PTSD]); or
ii) Compares women with disability to men with disability; or
iii) Only focuses on violence experienced before the age of 15; or
iv) Utilizes data from case studies or client files;
v) Is based on caregiver report and/ or forensic exam;
vi) Focuses on validity/ reliability of the measure or scale development.

These exclusion criteria were developed to ensure that the identified literature addresses the specific study aims and identify a body of literature that allows for contrast and comparison to answer the key research questions.

Mental disorders are often considered a part of disability. However, specifically in the area of violence against women, there is a robust evidence-base concerning the associations between common mental disorders (depression, anxiety and PTSD) and violence against women including several systematic reviews and meta analyses [27] [28] [29]. Given the aim of this scoping review to focus on the an area of measurement and methodology that is far less well-developed, we are limiting the breadth of our scoping review by excluding studies focusing solely on common mental disorders.

For peer review only - http://bmjopen.bmj.com/site/about/guidelines.xhtml

We will identify characteristics of studies (published and grey literature) meeting inclusion criteria, with a focus on mapping and evaluating measures of violence used in this research, identifying types of violence assessed, instruments utilized, and specifics of measures (i.e. perpetrator, time frame). This review differs from previous reviews of violence against persons with disability by focusing on: i) women, ii) any setting (community, institution, for example), iii) any type of violence and perpetrator, iv) measurement of disability (which measures, how measured), and iv) measurement of violence (which measures, how measured).

Data management

EndNote V.X9 will be used as our bibliographic software management platform. We will remove duplicates using EndNote, prior to exporting titles and abstracts to an Excel spreadsheet for review. Data extraction results will be recorded in separate Excel spreadsheets. A flow diagram will be presented in any final publications, showing results of each stage of the review and adhering to the PRISMA statement.

Selection of studies

Two authors will independently review titles and abstracts retrieved through the search strategy, to determine which should be included for full text review. If an abstract or title is considered relevant by either of the authors, it will be included for full text review. Two authors will independently review all articles selected for full text review for eligibility, to reach consensus on inclusion in the review. Any discrepancies will be resolved with the input of the third team member. Reasons for excluding articles will be recorded.

Data extraction

After full text review, the following data will be extracted from all included articles using a standardized data extraction form: country studied; research questions; study design (comparing individuals with disability vs. people without disability); sampling method

For peer review only

and sample characteristics; data collection (measurement method); disability measurement (definitions, measurement [self-report, instrument], measure of severity, functional impairment); violence measurement (definition, types measured, perpetrators, time frame, instruments use); data analysis methods; risk and protective factors; main findings (as reported by the study's own authors); ethical considerations and discussion of disability and violence specific issues; and any reported study limitations. The data extraction tool was designed specifically for this scoping review, and as such, includes necessary variables to address the aims of the study.

Data extraction will be conducted independently by one author (SM), and accuracy of the data extraction checked by a second author, with discrepancies resolved by discussion and, if necessary, by discussion with another author (CGM) to reach consensus. Reviewers will develop and pre-test a data extraction spreadsheet, to be used to compile a summary of characteristics and key findings of the included studies. The spreadsheet will also include categories relevant to data synthesis, described further below. We will not conduct quality assessment, given this is a scoping review. Pham et al. (2014) note that one of the distinctions between a systematic review and scoping review is that a scoping review aims to describe available material without critical appraisal of studies, and therefore, quality assessment is less necessary and common in scoping reviews [30]. This data extraction process corresponds with Stage 4, Charting the data, in Arksey and O'Malley's framework.

Data synthesis

We will present results of the search and data extraction, using both simple quantitative summaries (i.e. tabulation of % of studies from each region, % of studies that utilized specific sampling methods), and narrative synthesis of the studies, which includes highlighting similarities and differences in the measures of disability and of violence employed in the included studies, and exploration of other patterns in aspects of study design and measurement methodologies in included studies [31]. This phase corresponds

For peer review only - http://bmjopen.bmj.com/site/about/guidelines.xhtml

with Arksey and O'Malley's Stage 5, Collating, summarizing and reporting the results. Figure 1 displays all components of the study process.

Patient and public involvement:

Patients were not involved in the development of this scoping review. Members of the public were not consulted specifically for the development of the research questions, however, previous research and consultations with experts has indicated that this is a relevant and important area of enquiry in the field of violence against women research.

Discussion:

This manuscript describes a protocol for a scoping review of global measurement of violence against women within the context of disability-focused research and vice versa. Strengths of the review include a rigorous and expansive search strategy, including disability and violence against women studies conducted by National Statistical Offices and not published in peer-reviewed literature; a clear and structured process of data extraction; and a focus on generating a map of available measures and methodologies assessing the intersection of violence and disability, within a body of evidence that has not been rigorously reviewed. The scoping review is designed to assess global literature, explicitly using search methods to ensure that studies conducted in low and middle-income countries are included. This will contribute to the discussion on improving the ways of measuring the intersections of disability and violence against women.

Improved understanding and expanded evidence on how disability and violence against women intersect is needed to develop effective evidence-based programming and policy for prevention and response to violence against women globally. Various aspects of lived experiences of disability may influence types of violence experienced, access to and utilization of services, and ways in which research, policy and programming on violence against women can be adapted or refined to adequately address the needs of women with disability who experience violence. Yet, as a report from a recent expert

For peer review only

consultation on measurement of violence against women with disabilities noted, "far more work needs to be done to establish appropriate, effective, agreed and internationally comparable methods for measuring many of the structural, institutional and interpersonal forms of violence that women with disabilities experience on a daily basis"[19]. Expanding understanding of linkages and intersections between disability and violence against women requires further consideration of how disability and violence are currently being assessed, including what type(s) of measures are being used and within what type(s) of methodologies and study designs.

A primary limitation is the definition of disability that is operationalized in the review. The World Health Organization recognizes that disability includes "impairments, activity limitations, and participation restrictions," and that disability "is a complex phenomenon, reflecting the interaction between features of a person's body and features of the society in which he or she lives" (https://www.who.int/topics/disabilities/en/). According to this definition, knowledge of the environment in which a person lives is critical to understanding someone's experience of disability. However, to keep the scope of the review manageable and with the understanding that research on the environmental component of disability is often lacking, we will utilize search terms for disability that primarily focus on impairments or specific health conditions that are known to cause particular impairments. This may limit the literature identified and included in the search and bias results towards research focused on only the bodily, health or impairment component of disability. A limitation of the process of study screening and selection is that one team member will conduct the grey literature search and identification process. This may limit the rigor of the grey literature search process, but will also enable review of more grey literature for possible inclusion (i.e. 10 pages of results for 3 separate searches). A limitation of the study design is the focus on quantitative literature. Qualitative descriptions are important components of understanding women's experiences of disability and violence. However, the focus of this scoping review is specifically on quantitative

For peer review only - http://bmjopen.bmj.com/site/about/guidelines.xhtml

measurement. This is motivated by the existing state of the field of evidence and need for data to answer key questions to inform prevention and response in violence against women policy and programs. A separate review of qualitative literature could complement the current study.

Ethics and dissemination:

Findings of the review will be used to inform recommendations regarding evidence-generation on disability and violence against women. Findings will be with shared researchers, practitioners, data users and generators with an interest in violence against women. We will also share results with members of the Technical Advisory Group to the Interagency Working Group on Violence against Women Estimation and Data, a group of experts on measurement of violence against women and global violence against women data convened by the Department of Sexual and Reproductive Health and Research, WHO. Final outcomes will be presented in a manuscript for publication in a peer-reviewed journal. This will be disseminated through the Interagency Working Group above and other partners. The Sensory Functions, Disability and Rehabilitation Team will also disseminate through their networks focused on disability, including through the Interagency Support Group for the Convention on the Rights of Persons with Disabilities. Dissemination will also engage with disability advocacy groups, through the International Disability Alliance.

For peer review only - http://bmjopen.bmj.com/site/about/guidelines.xhtml

Figure 1: Study Process

Contribution: SM and CGM designed and developed the scoping review, which was conceptualized by CGM. SM, ML and LL developed and refined search strategies with input from CGM. SM prepared the manuscript with substantive input from all other authors. All authors reviewed the manuscript prior to submission.

Competing interest: There are no competing interests.

Funding: The scoping review will be conducted with funding from the Department for International Development for the UN Women-World Health Organization Joint Programme on Strengthening Methodologies and Measurement and building national capacities for violence against women data through the UNDP-UNFPA-UNICEF-WHO-World Bank Special Programme of Research, Development and Research Training in Human Reproduction (HRP), a cosponsored programme executed by the World Health Organization (WHO).

For peer review only

1. Gibbs A, Duvvury N, Scriver S. What Works Evidence Review: The relationship between poverty and intimate partner violence. In: What Works to Prevent Violence, ed., 2017.
2. Heise LL, Kotsadam A. Cross-national and multilevel correlates of partner violence: an analysis of data from population-based surveys. *The Lancet Global health* 2015;3(6):e332-40. doi: 10.1016/s2214-109x(15)00013-3 [published Online First: 2015/05/24]
3. Abramsky T, Watts CH, Garcia-Moreno C, et al. What factors are associated with recent intimate partner violence? findings from the WHO multi-country study on women's health and domestic violence. *BMC public health* 2011;11:109. doi: 10.1186/1471-2458-11-109 [published Online First: 2011/02/18]
4. Hughes K, Bellis MA, Jones L, et al. Prevalence and risk of violence against adults with disabilities: a systematic review and meta-analysis of observational studies. *Lancet (London, England)* 2012;379(9826):1621-9. doi: 10.1016/s0140-6736(11)61851-5 [published Online First: 2012/03/02]
5. Brownridge DA, Ristock J, Hiebert-Murphy D. The high risk of IPV against Canadian women with disabilities. *Medical science monitor : international medical journal of experimental and clinical research* 2008;14(5):Ph27-32. [published Online First: 2008/04/30]
6. Copel LC. Partner abuse in physically disabled women: a proposed model for understanding intimate partner violence. *Perspectives in psychiatric care* 2006;42(2):114-29. doi: 10.1111/j.1744-6163.2006.00059.x [published Online First: 2006/05/09]
7. United Nations Development Programme. What does it mean to leave no-one behind? A UNDP discussion paper and framework for implementation. New York: UNDP, 2018.
8. Dunkle K, van der Heijden I, Stern E, et al. Disability and Violence against Women and Girls: Emerging Evidence from the What Works to Prevent Violence against Women and Girls Global Programme. In: Works W, ed., 2018.
9. Bowen E, Swift C. The Prevalence and Correlates of Partner Violence Used and Experienced by Adults With Intellectual Disabilities: A Systematic Review and Call to Action. *Trauma, violence & abuse* 2017:1524838017728707. doi: 10.1177/1524838017728707 [published Online First: 2018/01/16]
10. Hughes RB, Lund EM, Gabrielli J, et al. Prevalence of interpersonal violence against community-living adults with disabilities: a literature review. *Rehabilitation psychology* 2011;56(4):302-19. doi: 10.1037/a0025620 [published Online First: 2011/11/30]
11. Plummer SB, Findley PA. Women with disabilities' experience with physical and sexual abuse: review of the literature and implications for the field. *Trauma, violence & abuse* 2012;13(1):15-29. doi: 10.1177/1524838011426014 [published Online First: 2011/11/11]
12. Mont D. Measuring health and disability. *Lancet (London, England)* 2007;369(9573):1658-63. doi: 10.1016/s0140-6736(07)60752-1 [published Online First: 2007/05/15]

For peer review only

13. Trani JF, Babulal GM, Bakhshi P. Development and Validation of the 34-Item Disability Screening Questionnaire (DSQ-34) for Use in Low and Middle Income Countries Epidemiological and Development Surveys. *PloS one* 2015;10(12):e0143610. doi: 10.1371/journal.pone.0143610 [published Online First: 2015/12/03]
14. Madans JH, Loeb M. Methods to improve international comparability of census and survey measures of disability. *Disability and rehabilitation* 2013;35(13):1070-3. doi: 10.3109/09638288.2012.720353 [published Online First: 2012/10/02]
15. Mactaggart I, Kuper H, Murthy GV, et al. Measuring Disability in Population Based Surveys: The Interrelationship between Clinical Impairments and Reported Functional Limitations in Cameroon and India. *PloS one* 2016;11(10):e0164470. doi: 10.1371/journal.pone.0164470 [published Online First: 2016/10/16]
16. Sabariego C, Oberhauser C, Posarac A, et al. Measuring Disability: Comparing the Impact of Two Data Collection Approaches on Disability Rates. *International journal of environmental research and public health* 2015;12(9):10329-51. doi: 10.3390/ijerph120910329 [published Online First: 2015/08/27]
17. The Asia Foundation. Understanding violence against women and children in Timor-Leste: Findings from the Nabilan Baseline Study – Main Report. Dili The Asia Foundation, 2016.
18. UNFPA, National Statistics Office of Mongolia. 2017 National Study on Gender-based Violence in Mongolia. Ulaanbaatar, 2018.
19. Vaughan C, Devine A, Diemer K, et al. Measuring the Prevalence of Violence against Women with Disabilities: kNOwVAWdata Initiative, 2018.
20. Vaughan C, Devine A, Ignacio R. Building capacity for a disability-inclusive response to violence against women and girls: experiences from the W-DARE project in the Philippines. *Gender & Development* 2016;24(2):245-60.
21. Shah S, Tsitsou L, Woodin S. Hidden Voices: Disabled Women's Experiences of Violence and Support Over the Life Course. *Violence against women* 2016;22(10):1189-210. doi: 10.1177/1077801215622577 [published Online First: 2016/01/15]
22. United Nations. Declaration on the elimination of violence against women. New York 1993.
23. WHO. Towards a Common Language for Functioning, Disability and Health: ICF. Geneva: WHO, 2002.
24. Arksey H, O'Malley L. Scoping studies: towards a methodological framework. *Int J Soc Res Methodol* 2005;8:19-32.
25. Colquhoun HL, Levac D, O'Brien KK, et al. Scoping reviews: time for clarity in definition, methods, and reporting. *Journal of clinical epidemiology* 2014;67(12):1291-4. doi: 10.1016/j.jclinepi.2014.03.013 [published Online First: 2014/07/19]
26. Peters MD, Godfrey CM, Khalil H, et al. Guidance for conducting systematic scoping reviews. *International journal of evidence-based healthcare* 2015;13(3):141-6. doi: 10.1097/xeb.0000000000000050 [published Online First: 2015/07/03]

For peer review only - http://bmjopen.bmj.com/site/about/guidelines.xhtml

27. Trevillion K, Oram S, Feder G, et al. Experiences of domestic violence and mental disorders: a systematic review and meta-analysis. *PloS one* 2012;7(12):e51740. doi: 10.1371/journal.pone.0051740 [published Online First: 2013/01/10]
28. Sparrow K, Kwan J, Howard L, et al. Systematic review of mental health disorders and intimate partner violence victimisation among military populations. *Social psychiatry and psychiatric epidemiology* 2017;52(9):1059-80. doi: 10.1007/s00127-017-1423-8 [published Online First: 2017/07/28]
29. Howard LM, Oram S, Galley H, et al. Domestic violence and perinatal mental disorders: a systematic review and meta-analysis. *PLoS medicine* 2013;10(5):e1001452. doi: 10.1371/journal.pmed.1001452 [published Online First: 2013/06/01]
30. Pham MT, Rajić A, Greig JD, et al. A scoping review of scoping reviews: advancing the approach and enhancing the consistency. *Research synthesis methods* 2014;5(4):371-85. doi: 10.1002/jrsm.1123 [published Online First: 2015/06/09]
31. Ryan R. Cochrane Consumers and Communication Review Group: data synthesis and analysis: Cochrane Consumers and Communication Review Group 2013.

For peer review only

For peer review only - http://bmjopen.bmj.com/site/about/guidelines.xhtml

Figure 1: Study process

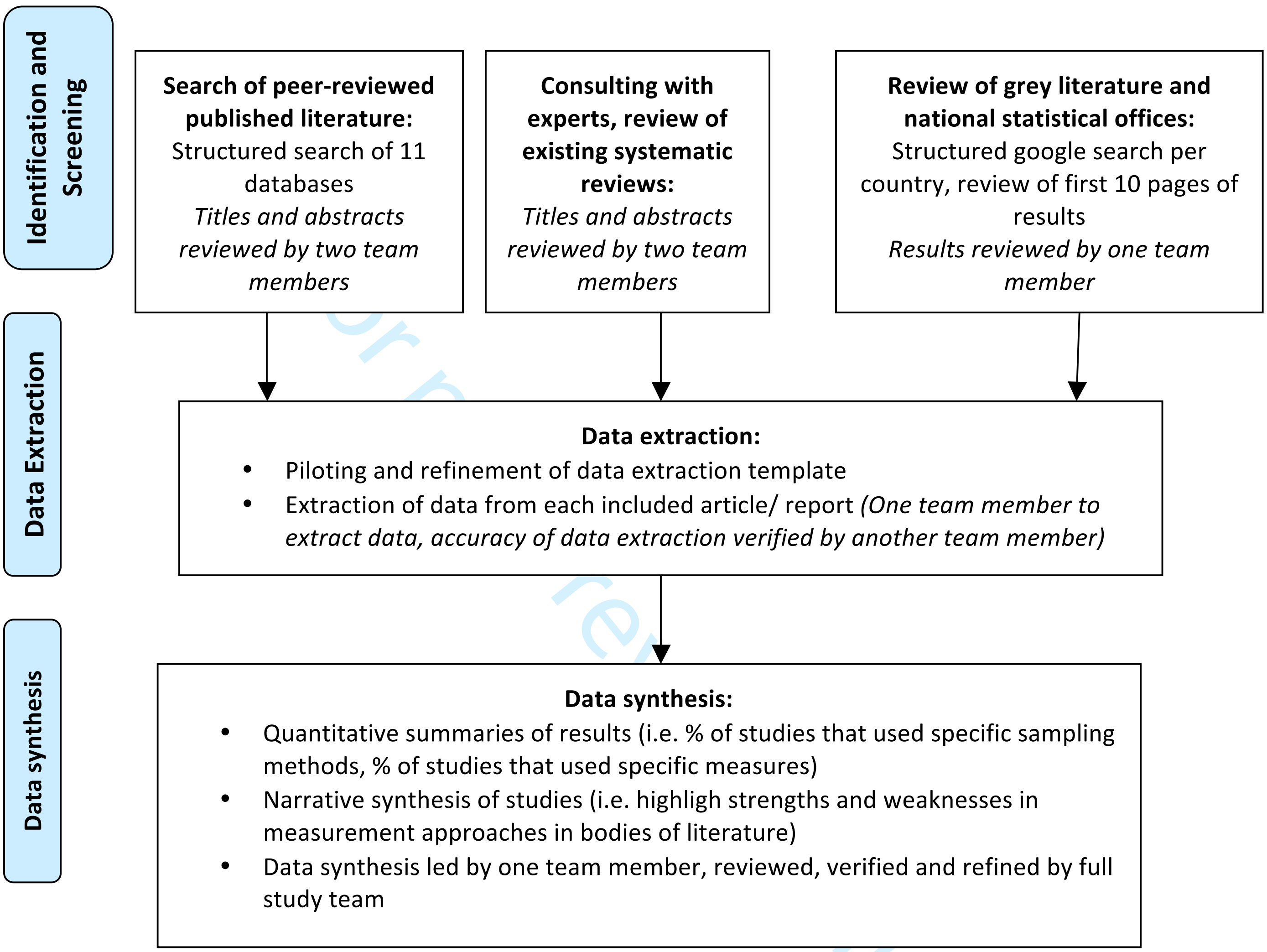

Supplement: Author's manuscript [file bmjopen-2020-040104.draft_revisions.pdf]
